# circRNA 001306 enhances hepatocellular carcinoma growth by up‐regulating CDK16 expression via sponging miR‐584‐5p

**DOI:** 10.1111/jcmm.16047

**Published:** 2020-11-01

**Authors:** Qian Liu, Cixiao Wang, Zeyou Jiang, Shan Li, Fang Li, Hua‐Bing Tan, Su‐yang Yue

**Affiliations:** ^1^ Department of Clinical Laboratory Hospital of Chengdu University of Traditional Chinese Medicine Chengdu China; ^2^ Nephrology Department 2 Hospital of Chengdu University of Traditional Chinese Medicine Chengdu China; ^3^ Department of Infectious Diseases and Lab of Liver Disease Renmin Hospital Hubei University of Medicine Shiyan China; ^4^ Department of Gastroenterology The Second People’s Hospital of Huai’an The Affiliated Huai’an Hospital of Xuzhou Medical University Huaian China

**Keywords:** CDK16, circ‐001306, hepatocellular carcinoma, miR‐584‐5p

## Abstract

Circular RNAs (circRNAs) have been demonstrated to play important roles in cancer progress. However, the roles in hepatocellular carcinoma (HCC) are still unclear. Here, we found has_circRNA_001306 (circ_1306) was up‐regulated in HCC tissues and cell lines. Knockdown the expression circ_1306 significantly suppressed HCC cell proliferation and induced the cell apoptosis in vitro and in vivo. Furthermore, we identified circ_1306 could up‐regulate the expression of CDK16 by sponging miR‐584‐5p. The expression of miR‐584‐5p was decreased, and the expression of CDK16 was increased in HCC tissues and cell lines. Meanwhile, either knockdown of miR‐584‐5p or overexpression of CDK16 could suppress the HCC cell proliferation. In vivo, overexpression of miR‐584‐5p or knockdown of circ_1306 could inhibit the expression of CDK16, and suppress tumour growth. Altogether, our findings suggested that circ_1306 could promoter HCC progress by miR‐584‐5p/CDK16 axis, which provided a novel marker for HCC diagnosis and treatment.

## 1 INTRODUCTION

Liver cancer is the most common malignant tumour over the world. The incidence is rising faster than that for any other cancer in both men and women, and the 5‐year survival rate is less than 18%.^1^ Hepatocellular carcinoma (HCC) is a type of primary liver cancer, which is accounting for approximately 90% of liver cancer. Most HCC patients were diagnosed at the advanced stages, missing the best treatment time, which seriously affected the survival time of patients.^2^ Thus, finding the method or marker for early diagnosis is important for prognosis of HCC.

Circular RNAs (circRNAs) have been reported as a regulator in many cellular processes, such as proliferation and differentiation.^3^ circRNAs is a class of noncoding RNAs with a characteristic of circle structure to prevent circRNAs from being degraded by endogenous RNase, therefore stably exist in cells. circRNAs regulated gene expression by sponging mircoRNAs (miRNA), RNA‐binding protein (RBP) sequestering agents and transcription regulators.^4^ Increasing studies showed that circRNAs play important roles in tumour procession, such as osteosarcoma cell^5^, leukaemia^6^, lung cancer^7^ and cervical cancer.^8^ Thus, circRNAs might be suitable as potential biomarkers and targets for novel therapeutic approaches for human disease. Many studies also suggested that circRNAs might involve in the process of hepatocellular carcinoma.^9^, ^10^, ^11^

Aim to find a new circRNA associated with the process of HCC, and this study analysed the circRNA expression profile in HCC in GEO database (GSE94508 ^12^and found one of the up‐regulated circRNAs, hsa_circRNA_001306 (circ_1306 for short). The purpose of this study was to explore the potential mechanism of circ_1306, its target miRNAs (hsa‐miR‐584‐5p, miR‐584‐3p for short) and CDK16 in the process of HCC.

## 2 MATERIALS AND METHODS

### 2.1 Cell culture and transfection

The human normal liver cell line (L02, iCell‐h054) and HCC cell lines SK‐HEP‐1 (iCell‐h190), Hep G2 (iCell‐h092), Huh‐7 (iCell‐h080) and Hep 3B (iCell‐h091) were obtained from icellbioscience (Shanghai, China). L02 cells were cultured in Roswell Park Memorial Institute (RPMI) 1640 medium (Life Technologies, Carlsbad, CA, USA) supplemented with 10% foetal bovine serum (FBS, Life Technologies). All HCC cells were cultured in Dulbecco's modified Eagle's medium (DMEM, Life Technologies) supplemented with 10% FBS. All cells were maintained in a humidified atmosphere of 5% CO_2_.

siRNA for circ_1306 (si‐1306), siRNA for negative control (si‐NC), miR‐584‐5p mimics (miR mimics), miR‐584‐5p inhibitor (miR‐inhibitor), negative control (miR‐NC) and CDK16 overexpression vector (OV‐CDK16) were obtained from GenePharma (Shanghai, China). The oligonucleotides and plasmids were transfected into cells using lipofectamine 2000 (Invitrogen, Waltham, MA, USA) according to the manufacturer's instructions.

### 2.2 qPCR

Total RNA of cells and tissues was extracted by TRIzol reagent (Invitrogen) according to the manufacturer's instructions. For Rnase R digestion assay, total RNAs were cultured with RNase R at 37°C for 15 minutes. The nuclear and cytoplasmic fractions were isolated using PARISTM Kit (Thermo Fisher Scientific, Carlsbad, CA, USA) following the manufacturer's instruction. RNAs were reverse‐transcript to cDNAs using RevertAid First Strand cDNA Synthesis Kit (Thermo Fisher Scientific) according to the manufacturer's protocol. QPCR were performed by SYBR™ Green PCR Master Mix using ABI PRISM 7500 Sequence Detection System (Life Technologies). The relative expression of circRNA was normalized by GAPDH, and the relative expression of miRNA was normalized by U6. The data were analysed by the method of 2^−ΔΔCt^.^13^ The primers for revers transcription and qPCR were obtained from Sangon Biotech (Shanghai, China).

### 2.3 Cell viability

Cell viability was analysed by cell counting kit‐8 (CCK‐8, Beyotime Biotechnology, Hangzhou, China). Cells (5000 cells/well) were seeded into 96‐well plates for 12 hours, then transfected with oligonucleotides and plasmids. The transfected cells were cultured for 48 hours, then incubated with 10 μL CCK‐8 reagents for 1 hours. The absorbance at 450 nm was measured by a microplate reader (Tecan, Switzerland).

### 2.4 Colony formation

The transfected cells were seeded into 6‐well plates at the concentration of 2000 cell per well and incubated for 14 days. The cells were fixed with methanol and stained by 0.1% crystal violet solution. The colonies were counted under the microscope. Every well counted three fields.

### 2.5 Cell apoptosis

Cell apoptosis was assayed by flow cytometry assay. Cells were seeded into 6‐well plates and transfected for 48 hours. Subsequently, cells were digested and resuspended in binding buffer to prepare single cell suspensions and stained using the annexin V‐FITC reaction reagent (BD Biosciences, NJ, USA) in the dark at room temperature for 30 minutes. Then, the stained cells were analysed by BD Accuri C6 Plus (BD Biosciences). The results were analysed using FlowJo v10.5 software.

### 2.6 Dual‐luciferase reporter assay

The sequence of circ_1306 (WT‐1306), mutated circ_1306 (Mut‐1306), the sequence of CDK16 3’‐UTR (WT‐CDK16) and mutated sequence of CDK16 3’‐UTR (Mut‐CDK16) were cloned and inserted into the psiCHECK‐2 vector (Promega, WI, USA). WT‐1306 or Mut‐1306 was co‐transfected into cells with miR‐584 or miR‐NC. WT‐CDK or Mut‐CDK was co‐transfected into cells with miR‐584 or miR‐NC. Dual‐luciferase assay system (Promega) was used to detect luciferase activity after transfection for 48 hours.

### 2.7 Western blot assay

Total proteins in tissues or cells were extracted with RIPA cell lysate (Beyotime) supplemented with PMSF (Beyotime) and quantified with Bradford protein assay (Beyotime). The extracted protein was separated by 12% SDS‐PAGE gel and transferred to PVDF membrane. After blocked with 5% BSA, the membrane was incubated with the GAPDH antibody (14485‐1‐AP, Proteintech, IL, USA), CDK16 antibody (ab154567, Abcam, Cambridge, MA, USA), Bcl‐2 antibody (ab182858, Abcam), Bad antibody (ab62465, Abcam), Cleaved‐Caspase‐3 antibody (ab2302, Abcam) or Cleaved‐Caspase‐9 antibody (ab2324, Abcam) under 4°C overnight. Then the washed membrane was incubated with the second antibody at room temperature for 1 hours. The band was exposed by ECL solution (Thermo Fisher Scientific) and analysed by Quantity One software (Bio‐Rad, San Diego, CA, USA).

### 2.8 Xenograft model

Hep 3B cells (1 × 10^6^) were injected subcutaneously into 6‐week‐old male BALB/c nude mice (National Rodent Laboratory Animal Resource, Shanghai, China). To establish xenograft tumour model, 18 male nude mice were randomly divided into 3 groups (n = 6 per group). After tumour volume reached more than 100 mm^3^, the NC, miR mimics and si‐1306 were injected. Tumour volume was measured by calipers and calculated as 0.5 × length ×width. After 30 days of modelling, the mice were sacrificed. The tumour tissues were separated and weighed. The expression of Ki67 was determined by immunohistochemistry (IHC). The cell apoptosis in tumour tissues was determined by Tunel assay (Beyotime).

### 2.9 Statistical analysis

The results were expressed as mean ± SD. The differences between the experimental and control groups were compared using t test. All statistical analyses were performed using GraphPad Prism 8.0 software. *P < *0.05 was considered statistically significance.

## 3 RESULTS

### 3.1 Circ_1306 was up‐regulated in HCC tissues and cell lines

**FIGURE 1:**
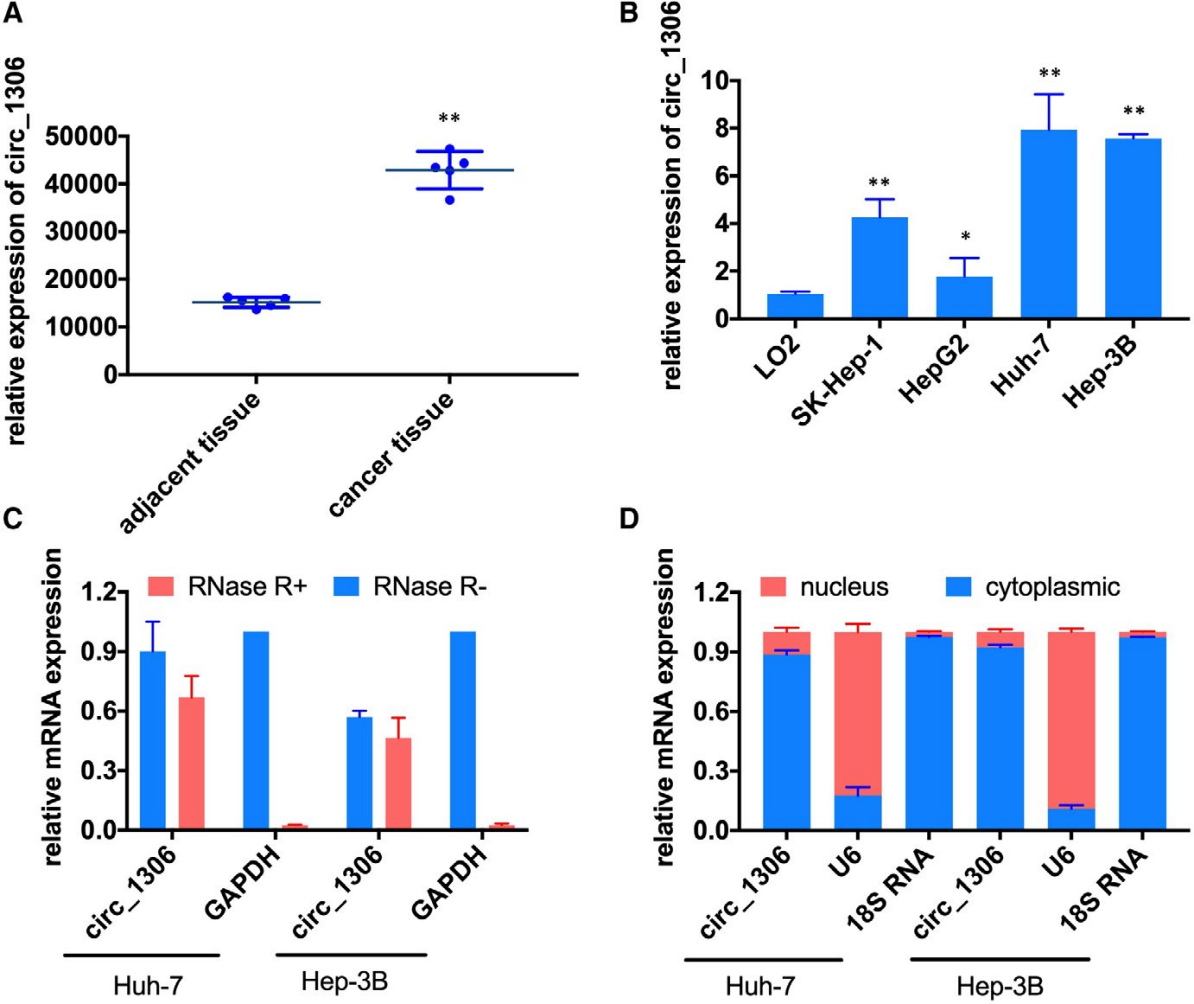
The relative expression of circ_1306 in HCC tissues and cell lines. (A) The expression of circ_1306 in HCC tissues (n = 5) and adjacent normal tissues (n = 5) in GSE94508. **P < 0.01 compared to adjacent normal tissues. (B) The relative expression of circ_1306 in the human normal liver cell line (L02) and HCC cell lines (SK‐HEP‐1, Hep G2, Huh‐7, and Hep 3B) was determined by qPCR. *P < 0.05 and **P < 0.01 compared to L02. (C) The expression of circ_1306 and GAPDH in Huh‐7 and Hep 3B cells was tested by qPCR after RNase R treated or untreated. (D) The distribution of circ_1306 in Huh‐7 and Hep 3B cells was detected by a nuclear and cytoplasmic separation assay. All data represent the mean ± SD

The expression of circ_1306 in para cancerous and HCC tissues of 5 patients was analysed by the data of GSE94508.^12^ The results showed that circ_1306 was significantly up‐regulated in HCC tissues Figure 1A. Meanwhile, the expression of circ_1306 in HCC cell lines was also higher than that in normal liver cell lines (L02 cells) and was highest in Huh‐7 and Hep3B cells Figure 1B. Therefore, Huh‐7 and Hep3B cells were selected for further experiments. Further experiments showed circ_1306 expressed in the cytoplasmic Figure 1C and were resistant to RNase R digestion Figure 1D).

### 3.2 Effect of knockdown circ_1306 on HCC cell proliferation

**FIGURE 2:**
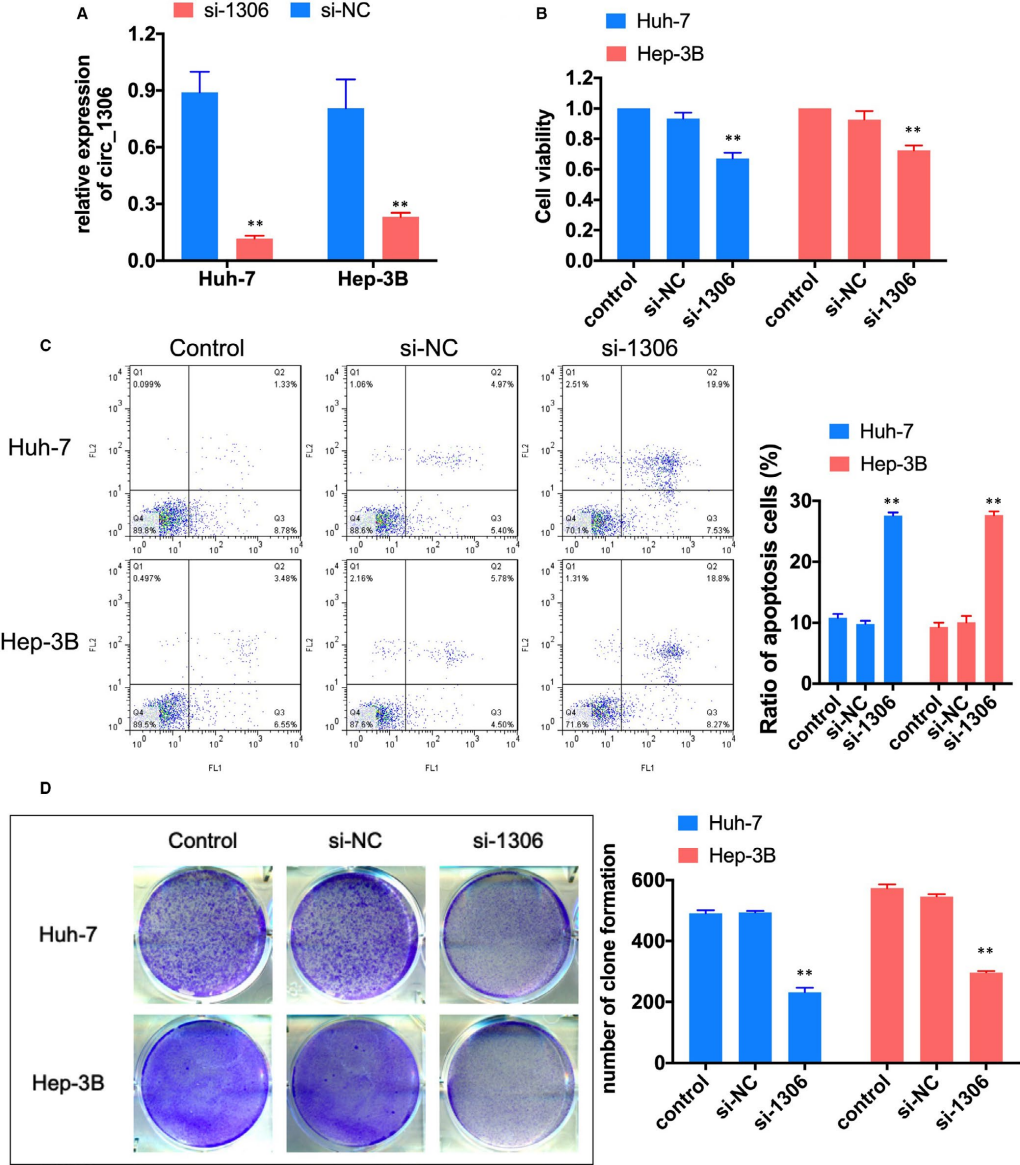
Knockdown of circ_1306 suppressed HCC cell proliferation. (A) The expression of circ_1306 in Huh‐7 and Hep 3B cells after si‐1306 or si‐NC transfection were tested by qPCR. (B) Cell viability was tested by CCK‐8 assay 48 h after si‐1306 or si‐NC transfection. (C) Cell apoptosis was analysed by flow cytometry assay 48 h after si‐1306 or si‐NC transfection. (D) Cell proliferation was tested by colony formation assay. All data represent the mean ± SD. **P < 0.01 compared to si‐NC group

To evaluate the effect of circ_1306 on HCC cell proliferation, the siRNA for circ_1306 (si‐1306) was transfected into Huh‐7 and Hep3B cells Figure 2A ^,^ and the cell proliferation was tested by CCK‐8 and colony formation. The results showed that knockdown of circ_1306 significantly reduced the cell activity and colony formation ability of Huh‐7 and Hep3B cells Figure 2B and D. Meanwhile, flow cytometry assay results showed that the ratio of apoptosis cells was significantly increased after si‐1306 transfection Figure 2C. These results suggested that knockdown circ_1306 could inhibit the cell proliferation and induce the cell apoptosis in HCC cells.

### 3.3 Circ_1306 served as a sponge of miR‐584‐3p

**FIGURE 3:**
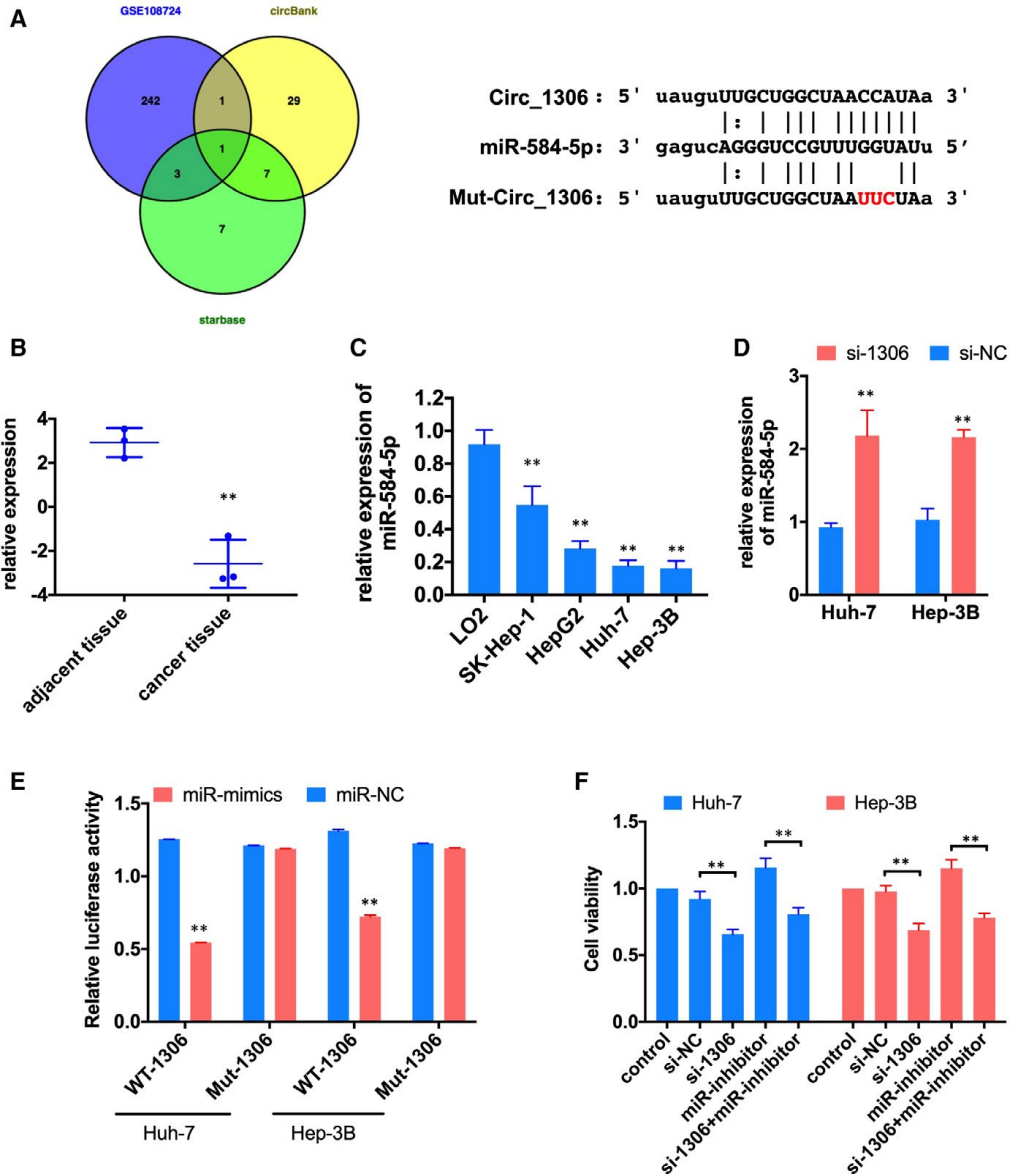
circ_1306 serves as a sponge for miR‐584‐5p. (A) A Venn drawing showed the overlapping of the target miRNAs of circ_1306 predicted by GSE108724, circBank and starbase. (B) The expression of miR‐584‐5p in HCC tissues (n = 3) and adjacent normal tissues (n = 3) in GSE108724. **P < 0.01 compared to adjacent normal tissues. (C) The relative expression of miR‐584‐5p in the human normal liver cell line (L02) and HCC cell lines (SK‐HEP‐1, Hep G2, Huh‐7 and Hep 3B) was determined by qPCR. **P < 0.01 compared to L02. (D) The expression of miR‐584‐5p in Huh‐7 and Hep 3B cells after si‐1306 or si‐NC transfection was tested by qPCR. **P < 0.01 compared to si‐NC group. (E) Dual‐luciferase reporter assay showed that miR mimics significantly reduced luciferase activity of WT‐1306 group. **P < 0.01 compared to miR‐NC group. (F) Cell viability was tested by CCK‐8 assay 48h after si‐1306, miR‐inhibitor or co‐transfection. **P < 0.01

**FIGURE 4:**
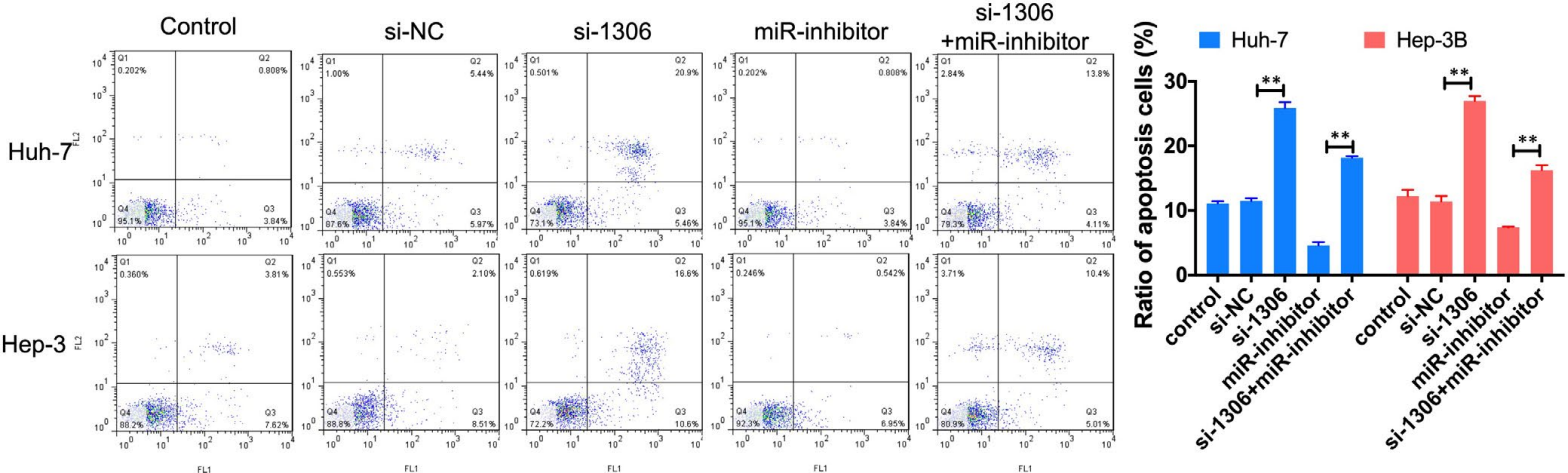
circ_1306 and miR‐584‐5p influenced the HCC cell apoptosis. Cell apoptosis was analysed by flow cytometry assay 48 h after si‐1306, miR‐inhibitor or co‐transfection. **P < 0.01. All data represent the mean ± SD

Previous studies suggested that circRNAs regulated gene expression by sponging miRNAs.^14^ We analysed the down‐regulated miRNAs in liver cancer tissue in the data of GEO (GSE108724 ^15^and predicted the potential target miRNAs of circ_1306 by circBank and starbase. The Venn diagram showed that miR‐584‐5p was only one potential target miRNA overlapping in the three databases Figure 3A ^,^ and the expression of miR‐584‐5p significantly reduced in liver cancer tissue Figure 3B. qRT‐PCR showed that the expression of miR‐584‐5p in HCC lines was also lower than that in normal liver cell lines (L02 cells), and was lowest in Huh‐7 and Hep3B cells Figure 3C. Dual‐luciferase reporter assay showed that miR‐584‐5p mimics significantly reduced luciferase activity of WT‐1306 group Figure 3D. Meanwhile, knockdown the expression of circ_1306 significantly induced the expression miR‐584‐5p in Huh‐7 and Hep3B cells Figure 3E. The CCK‐8 assay showed that inhibited the expression of miR‐584‐5p could significantly increase the proliferation of Huh‐7 and Hep3B cells, and this effect could recover by circ_1306 knockdown Figure 3F. Flow cytometry assay results showed that the ratio of apoptosis cells was significantly decreased after miR‐inhibitor transfection, and the ratio of apoptosis cells was increased after si‐1306 and miR‐inhibitor co‐transfection Figure 4. These results suggested that miR‐584‐5p was a target of circ_1306.

### 3.4 CDK16 was a direct target of miR‐584‐5p

**FIGURE 5:**
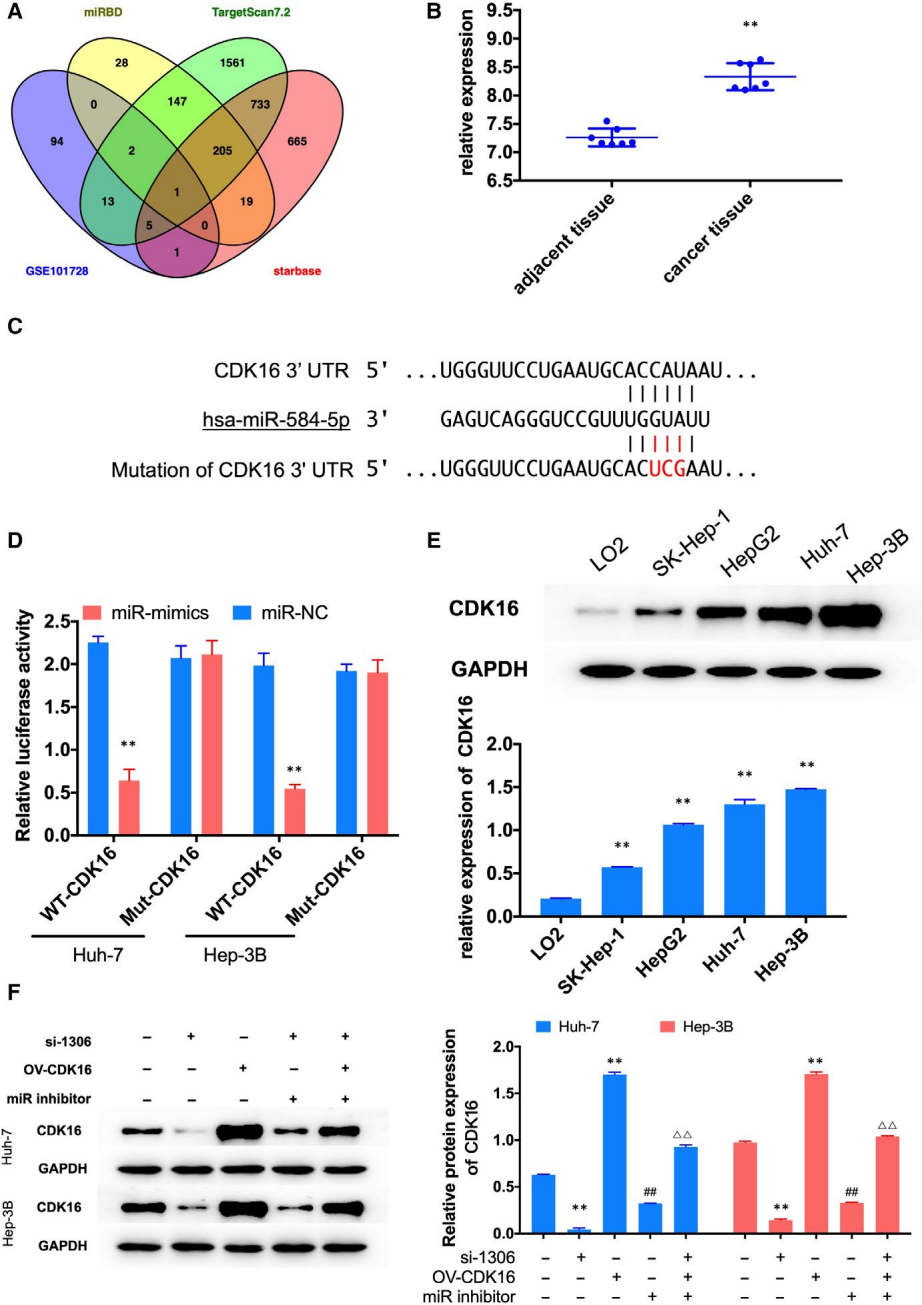
CDK16 was a direct target of miR‐584‐5p. (A) A Venn drawing showed the overlapping of the target genes of miR‐584‐5p predicted by GSE101728, miRDB, TargetScan and starbase. (B) The expression of CDK16 in HCC tissues (n = 7) and adjacent normal tissues (n = 7) in GSE108724. **P < 0.01 compared to adjacent normal tissues. (C) The binding site between miR‐584‐5p and CDK16 3’‐UTR. (D) Dual‐luciferase reporter assay showed that miR mimics significantly reduced luciferase activity of WT‐CDK16 group. **P < 0.01 compared to miR‐NC group. (E) The protein expression of CDK16 in the human normal liver cell line (L02) and HCC cell lines (SK‐HEP‐1, Hep G2, Huh‐7 and Hep 3B) were determined by Western blot. **P < 0.01 compared to L02. (F) The protein expression of CDK16 in Huh‐7 and Hep 3B cells after si‐1306, miR‐inhibitor, OV‐CDK16 or co‐transfection was tested by qPCR. **P < 0.01 compared to control group. ##*P* < 0.01 compared to si‐1302 group. ΔΔ*P* < 0.01 compared to OV‐CDK16 group. All data represent the mean ± SD

**FIGURE 6:**
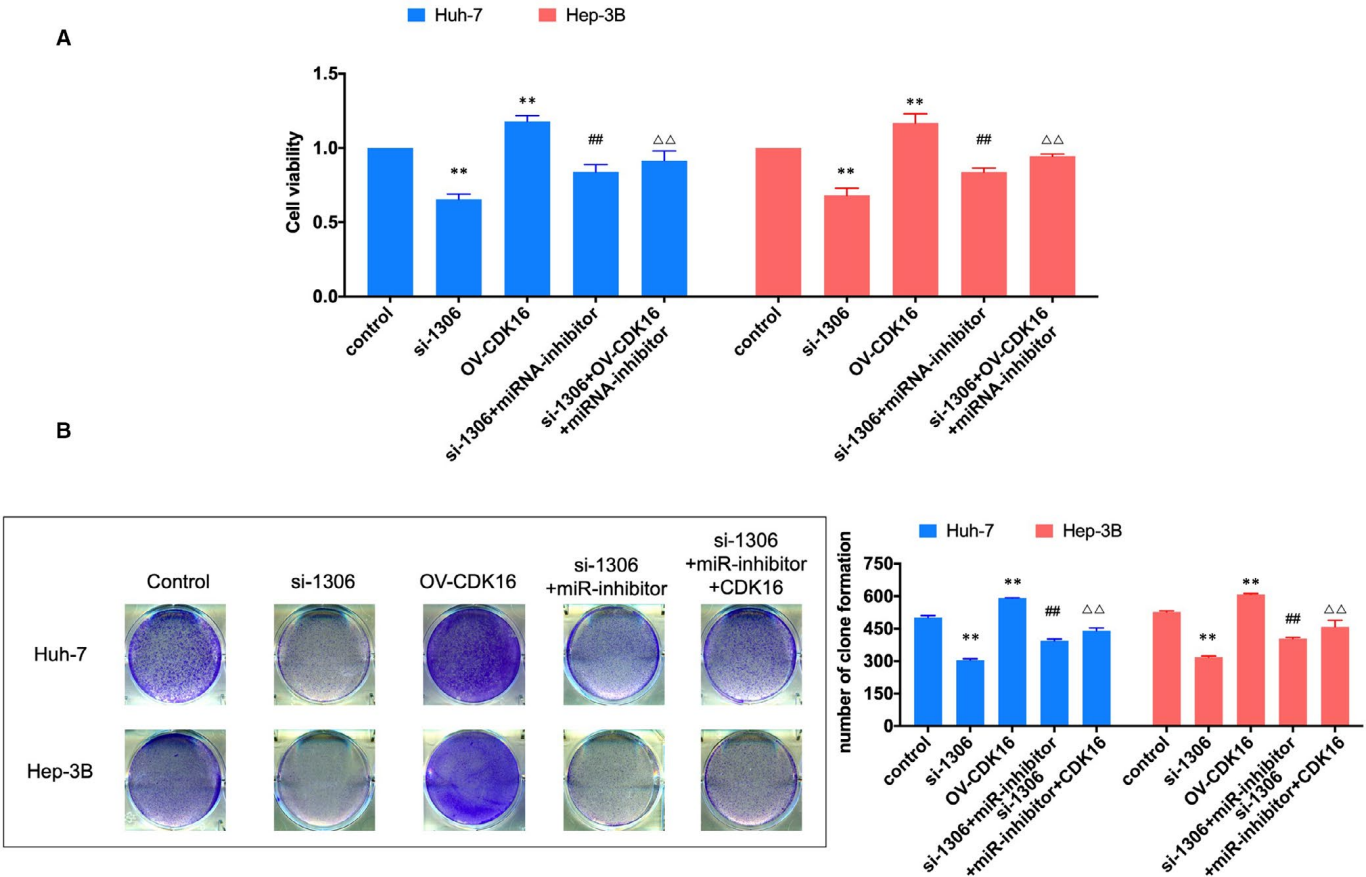
The effect of circ_1306/miR‐584‐5p/CDK16 on cell proliferation in HCC cell lines. (A) Cell viability was tested by CCK‐8 assay after transfection. (B) Cell proliferation was tested by colony formation assay. **P < 0.01 compared to si‐NC group

**FIGURE 7:**
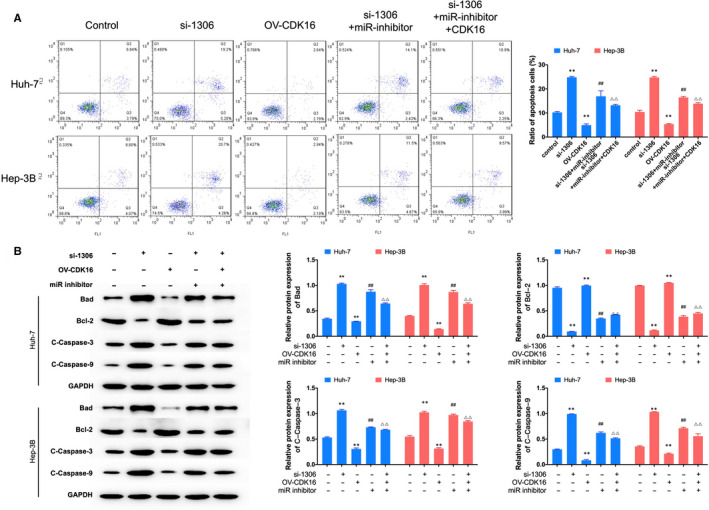
The effect of circ_1306/miR‐584‐5p/CDK16 on cell apoptosis in HCC cell lines. (A) Cell apoptosis was analysed by flow cytometry assay after transfection. **P < 0.01 compared to si‐NC group. (B) The protein expression of apoptosis‐related genes was treated by Western blot. **P < 0.01 compared to control group. ##*P* < 0.01 compared to si‐1302 gourp. ΔΔ*P* < 0.01 compared to OV‐CDK16 group. All data represent the mean ± SD

To find the target of miR‐584‐5p, we analysed the up‐regulated genes in liver cancer tissue in the data of GEO (GSE101728 ^16^ and predicted the potential target genes of miR‐584‐5p by miRDB, TargetScan and starbase. The Venn diagram showed that CDK16 was only one potential target miRNA overlapping in the four databases Figure 5A ^,^ and the expression of CDK16 significantly increased in liver cancer tissue Figure 5B. Western blot assay showed that the protein expression of CDK16 in HCC lines was increased in liver cancer cells Figure 5E. Dual‐luciferase reporter assay showed that miR‐584‐5p mimics significantly reduced luciferase activity of WT‐CDK16 group Figure 5C and D. Furthermore, the protein expression of CDK16 was increased by miR‐inhibitor transfection and decreased by si‐1306 transfection. The inhibiting effect of si‐1306 on the expression of CDK16 was recovered by miR‐inhibitor Figure 5E. CCK‐8 and colony formation assay showed that overexpression of CDK16 could induce cell proliferation, and this effect could recover by inhibit the expression of circ_1306 and miR‐584‐5p Figure 6. Flow cytometry assay results showed that the ratio of apoptosis cells was significantly decreased after CDK16 overexpression, and the ratio of apoptosis cells was increased after OV‐CDK16, si‐1306 and miR‐inhibitor co‐transfection Figure 7A. Western blot assay tested the protein expression of apoptosis‐related genes Figure 7B. The results showed that the expression of Bad, Cleaved‐Caspase‐3 and Cleaved‐Caspase‐9 was induced by circ_1306 inhibiting and reduced by CDK16 overexpression. The expression of Bcl‐2 was reduced by circ_1306 inhibiting and induced by CDK16 overexpression. The effect of CDK16 overexpression was recovered by si‐1306 and miR‐inhibitor transfection. These results suggested that CDK16 was a direct target of miR‐584‐5p and involved in the proliferation of liver cancer cells.

### 3.5 Suppressing the expression of circ_1306 reduced the tumour growth in vivo

**FIGURE 8:**
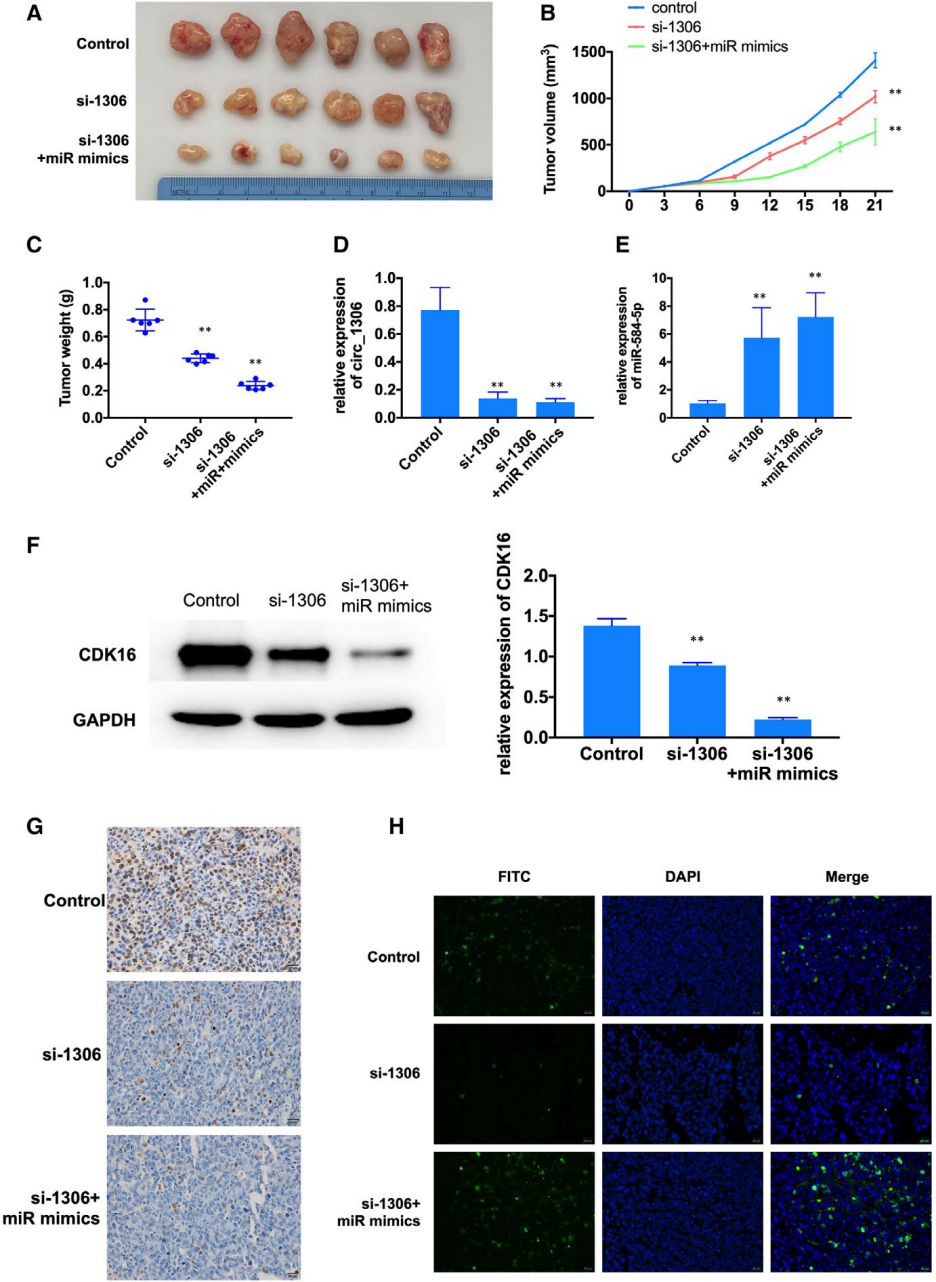
The effect of circ_1306/miR‐584‐5p/CDK16 on tumour growth in vivo. (A‐C) si‐1306 and miR mimics reduced xenograft growth in nude mice. (D‐F) QRT‐PCR and Western blot were used to explore circ_1306, miR‐584‐5p and CDK16 expression in tumour tissues. All data represent the mean ± SD. **P < 0.01 compared to si‐NC group. (G) The expression of proliferation marked (Ki67) in tumour tissues was tested by IHC. (H) The cell apoptosis in tumour tissues was tested by Tunel assay

We further examined the effect of circ_1306 on tumour growth in vivo. The results showed that inhibiting the expression of circ_1306 decreased tumour volume and weight than control group, and overexpression of miR‐584‐5p enhanced the effect of si‐1306 on tumour growth Figure 8A‐C. qRT‐PCR showed that suppressing the expression of circ_1306 induced the expression of miR‐584‐5p Figure 8D and E. Western blot showed that knockdown the expression of circ_1306 and overexpression of miR‐584‐5p reduced the expression of CDK16 Figure 8F. IHC assay showed the expression of proliferation marker (Ki67) was reduced by circ_1506 knockdown and miR‐584‐5p overexpression Figure 8G. Tunel assay showed the apoptosis cells were increased by circ_1506 knockdown and miR‐584‐5p overexpression Figure 8H. These results suggested that circ_1306/miR‐584‐5p/CDK16 involved in tumour growth in vivo.

## 4 DISCUSSION

Recently, increasing studies demonstrated that circRNAs involved in various cancers, including hepatocellular carcinoma.^9^, ^10^, ^11^, ^12^ However, the roles of circRNAs in the process of HCC are still unclear. In this study, we identified an up‐regulated named circ_001306 in HCC tissues in GEO database and analysed the mechanism of circ_1306 in HCC cell proliferation.

Similar to the results in previous data of HCC tissues (GSE94508), our results showed that circ_001306 was also highly expressed in HCC cell lines compared to human normal live cell lines. The knockdown assay showed that circ_1306 induced the proliferation and reduced the apoptosis in HCC cell lines. Furthermore, the tumour growth was decreased by circ_1306 knocking down in vivo. These results revealed that circ_1306 might serve as tumour promoter in HCC.

Increasing studies indicated that circRNAs play as miRNAs sponges to regulate gene expression and cell progress.^17^ In this study, we identified a potential target miRNA of circ_1306 by bioinformatics. miRNA‐584‐5p was down‐regulated in HCC tissues and cell lines. The expression of miR‐584‐5p is increased after circ_1306 knockdown, and dual‐luciferase reporter assay demonstrated the directly binding site between circ_1306 and miR‐584‐5p. The rescue assays showed that miR‐584‐5p inhibitor reversed the suppressive effects of circ_1306 siRNA on HCC cell proliferation. These results suggested that circ_1306 might regulate proliferation of HCC cell by sponging miR‐584‐5p. miR‐584‐5p has been demonstrated involving in the cancer progress in many studies.^18^, ^19^, ^20^

In this study, we also found that miR‐584‐5p could directly binding to the 3’‐UTR of CDK16. Furthermore, overexpression of CDK16 could induce cell proliferation, and this effect could recover by inhibit the expression of circ_1306 and miR‐584‐5p. CDK16 is a number of the cyclin‐dependent kinase (CDK) family.^21^ Recent study suggested that increased CDK16 expression was associated with lower overall survival in several types of cancer.^22^ CDK16 could suppress the expression and the stable of tumour suppressor p27, and down‐regulate the expression of c‐Myc.^23^, ^24^ These results indicated that circ_1306 might regulate cell proliferation by sponging miR‐584‐5p to induce the expression of CDK16. The precise mechanism of CDK16 in HCC needs more experiments to illuminate.

Altogether, this study demonstrated that circ_1306 could alleviate the inhibition effect of miR‐584‐5p on the expression of CDK16 by sponging miR‐584‐5p, thus promoting the tumour growth by inducing cell proliferation and reducing cell apoptosis. This study provides circ_001306/miR‐584‐5p/CDK16 as a new target for HCC diagnose and treatment.

## CONFLICT OF INTEREST

The authors declare no conflict of interest.

## AUTHOR CONTRIBUTIONS

**Qian Liu:** Conceptualization (lead); Data curation (equal); Software (equal); Validation (equal); Writing‐review & editing (equal). **Cixiao Wang:** Conceptualization (supporting); Formal analysis (equal); Software (equal); Validation (equal); Visualization (equal); Writing‐review & editing (lead). **Zeyou Jiang:** Investigation (lead); Methodology (equal); Resources (equal); Validation (equal); Writing‐original draft (equal); Writing‐review & editing (equal). **Shan Li:** Data curation (equal); Formal analysis (equal); Investigation (lead); Writing‐original draft (equal). **Fang Li:** Conceptualization (lead); Funding acquisition (equal); Supervision (lead). **Hua‐Bing Tan:** Funding acquisition (lead); Writing‐review & editing (equal). **Su‐yang Yue:** Funding acquisition (equal); Resources (equal); Supervision (equal).

## Data Availability

The data that support the findings of this study are available from the corresponding author upon reasonable request.
